# Kinase-associated gene mutation pattern and clinical relevance in 205 patients with core binding factor leukemias

**DOI:** 10.1038/bcj.2016.107

**Published:** 2016-11-11

**Authors:** Y-S Chen, P-P Wang, Y Hu, Y-M Zhu, B Chen, J-Y Huang, J-M Li, X-Q Weng, Y Yu, Y Shen

**Affiliations:** 1 Shanghai Institute of Hematology, Department of Hematology, Ruijin Hospital Affiliated to Shanghai Jiao Tong University School of Medicine, Shanghai, China

Core binding factor (CBF) leukemia represents an individual subgroup of the disease, which accounts for 20% of acute myeloid leukemia (AML), characterized by the special t(8;21)(q22;q22) translocation most in AML-M2 variant (CBFα leukemia) or inv(16)(p13q22)/t(16;16) rearrangement in AML-M4 with eosinophilia (CBFβ leukemia), respectively.^1^ Chimerical fusion genes *AML1-ETO* and *CBF-MYH11* are formed by these two cytogenetic changes, respectively, which finally lead to the leukemogenesis.^2^ Generally, CBF leukemias are considered to have favorable treatment outcome and prognosis and most centers regard CBF markers as ‘good' cytogenetic factor, with a 5-year overall survival (OS) rate over 50%.^3^ However, given using similar treatment strategy, such as ‘3+7' regimen in induction and high-dose Ara-C in consolidation, the treatment outcome of CBF leukemia in Chinese patients were not as good as reported by western groups.^4^ Interestingly, the incidence of CBFβ leukemia is even significantly lower than the western countries, as shown in our previous report; in 1185 AML patients, only 18 M4 with eosinophilia patients were identified. The difference of genetic background between Chinese and western population may be the reason, however, until now, evidence remains unavailable.^5^

In mouse model, stepwise leukemogenesis in AML with t(8;21)/*AML1-ETO* is proved by the phenomena that coexpression of *C-KIT* N822K and *AML1-ETO* induces the full development of AML, whereas single or *C-KIT* is not sufficient to lead to the leukemia. Similarly, transgenic mice of *CBF-MYH11* only induce a myeloid maturation block.^6^ Therefore, it could be concluded that additional mutations, especially kinase-associated mutations, providing a second ‘hit'^7^ may play a crucial role in the evolving of the disease.

In this study, we included 205 newly diagnosed AML patients, including 180 patients with CBFα and 25 patients with CBFβ leukemia, to investigate the potential role of additional mutations beyond *AML1-ETO* and *CBF-MYH11* in these diseases. All the patients received standard first-line treatment of DNR (daunorubicin), A (Ara-c(cytarabine))-like regimen. In the consolidation therapy, young patients were treated with high-dose cytarabine-based chemotherapy. Allogenetic stem cell transplantation was not used as first-line treatment in first time to complete remission. This study was approved by the ethnic board of the participating centers. All patients were given informed consent for both treatment and cryopreservation of bone marrow and peripheral blood according to the Declaration of Helsinki.

Genomic DNA and total RNA were extracted as previously reported.^8^ We had screened the mutational status of *FLT3*-ITD and -TKD, *C-KIT*, *N-RAS*, *CEPBA*, *WT1*, *ASXL1*, *DNMT3A*, *NPM1*, *MLL*, *IDH2* and *TET2* genes by distinct approaches. A chip-based matrix-assisted laser desorption/ionization time-of-flight mass spectrometry analysis system (iPLEXTM, Sequenom, San Diego, CA, USA) was used to assess the mutational status of *FLT3*-TKD, *N-RAS*, *NPM1*, *IDH1* and *IDH2*. For mutations of *FLT3*-ITD, and those in *C-KIT*, *CEPBA*, *WT1*, *ASXL1*, *DNMT3A* and *TET2* genes, samples were analyzed by whole-gene sequencing. Six *MLL*-related common fusion genes, including *MLL-AF9*, *MLL-AF10*, *MLL-AF6*, *MLL-ELL*, *MLL-ENL* and *MLL-AF17*, were detected via multiplex RT-PCR strategy. Briefly, all samples were screened with two parallel multiplex RT-PCR reactions. If there were positive PCR fragments in the samples, split-out PCR was performed to determine the fusion gene type. The fusion genes, such as *AML1(CBFα)-ETO* and *CBFβ-MYH11*, were determined by RT-PCR technique.

Kaplan-Meier and hazard ratio analyses were used to calculate and compare the OS and disease-free survival (DFS), respectively. Cox model was used for the multivariate analysis of associations of survival and potential prognostic factors.

The characteristics of the 205 patients with CBF AML, including 180 CBFα and 25 CBFβ, are summarized in Table 1. The incidence of CBFβ leukemia was significantly lower than CBFα leukemia, which was not concurrent with the western population, which might be contributed by the difference of genetic backgrounds between Chinese and Caucasian populations. In the 205 patients, 82 (40%) patients carried at least one mutation. *C-KIT* (55/205, 26.8%), and *N-RAS* (18/205, 8.8%) and *CEBPA* (10/205, 8.8%) mutations were identified as the most common additional gene mutations, whereas another kinase-associated gene, *FLT3* mutations were very few (2/205, 1.0%). When we combined *C-KIT*, *N-RAS* and *FLT3* mutation together as a group of class I mutation, 67 (32.7%) patients contained such events. Other mutations could also be identified in relative low incidence (Supplementary Table 1). Interestingly, in CBF leukemia, mutual coexistence could be observed among the class I mutation (Figure 1a). Seven patients carried *C-KIT* and *N-RAS* and one patient carried *C-KIT* and *FLT3* mutations together. We identified a similar incidence of *C-KIT* (30.2% and 13.6% in CBFα and CBFβ, respectively) and *N-RAS* (8.4% and 19.0% in CBFα and CBFβ, respectively), in contrast, *FLT3* was very few identified in our group, which is different with the western reports (around 10%) but similar with the Asian series (1%) (Supplementary Table 2). This distribution of gene mutations is totally converse to acute promyelocytic leukemia, which have higher incidence of *FLT3*, whereas *C-KIT* are few identified.^9^

As far as the possible association with clinic features, there was no significant difference regarding the age, gender and median WBC count in different mutation groups, with the exception of bone marrow blasts, which seemed higher in class I mutation group (*P*<0.001, Supplementary Table 3).

Among 205 CBF leukemia patients, 160 (78.0%) achieved CR. CBFβ leukemia seemed to have a higher CR rate as compared with CBFα leukemia, however, no significance was achieved (22/25 (88.0%) vs 138/180 (76.7%), *P*=0.302). No adverse effects of *FLT3*-ITD/TKD, *N-RAS*, *C-KIT* and class I mutations were observed on CR induction either in CBFα leukemia or in CBFβ leukemia (Supplementary Table 4). Hence, no further multivariate analysis was performed.

The OS of CBFβ leukemia was significantly higher than CBFα leukemia (median OS: 40.0 vs 18.0±1.1 months, *P*=0.030, median DFS: 21.0±5.5 vs 18.0±4.0 months, *P*=0.438, Supplementary Figures 1A and 1B).

In 180 CBFα leukemia patients, *C-KIT* and class I mutations were associated with poor OS and DFS, respectively. For *C-KIT* mutant (−) and (+) patients, the median OS and DFS was 20.2±2.2 and 14.0±3.5 months (*P*=0.050), and 25.0±10.0 and 14.0±1.4 months (*P*=0.046), respectively. And for class I mutant (−) and (+) patients, the median OS and DFS was 23.0±3.4 and 17.0±3.0 months (*P*=0.030), and 34.0±11.5 and 15.0±1.3 months (*P*=0.048), respectively. The Kaplan-Meier survival curves were shown in Figure 1b and c. No significance was observed in different *N-RAS* mutant subgroup (*P*=0.497 and 0.641, respectively), which might be caused by the small samples.

In 25 CBFβ leukemia patients, there is no significant difference between different mutant groups (OS: *P*=0.935, 0.379 and 0.848 for class I, *C-KIT* and *N-RAS*, respectively; DFS: *P*=0.562, not fit due to small sample, and 0.426 for class I, *C-KIT* and *N-RAS*, respectively).

In multivariate analysis of OS and DFS involving clinical parameters and class I mutation in 180 CBFα leukemia patients, class I mutation remained independent prognostic factor for OS (*P*=0.041), whereas no factor was the independent one for DFS (Supplementary Table 5).

CBF AML is a unique subtype of leukemia, whose diagnosis is so strongly upon detection of clonal genetic abnormalities of t(8;21)(q22;q22) and inv(16)(p13q22)/t(16;16), or their related gene fusion of *AML1-ETO* and *CBF-MYH11*, whereas the proportion of bone marrow blasts.^10^ It was reported that the incidence of CBFα and CBFβ AML was around 7% and 5–8%, respectively. Although lack of strong epidemiology data, our previous experience^11^ and this work proved that, in Chinese population, a slight more CBFα leukemia patients are presented, whereas CBFβ AML are very few. In this study, a similar incidence of *C-KIT* (30.2% and 13.6% in CBFα and CBFβ, respectively) and *N-RAS* (8.4% and 19.0% in CBFα and CBFβ, respectively) was identified; whereas *FLT3* was very few in our group, similar with the Asian reports.

CBF AML is considered to have favorable prognosis when received standard DNR (daunorubicin), A (Ara-c(cytarabine)) induction and high-dose Ara-C-based consolidation. However, in Chinese population, as presented in this study and previous reports, the treatment outcome was not as good as the western reports, given the similar treatment, especially for CBFα AML. Huang *et al.*^12^ even use allogenetic stem cell transplantation as the front-line treatment for t(8;21) AML patient, regarding the mutational status of *C-KIT* and minimal residual disease during the treatment. Our data proved that CBFβ AML have a better survival as compared with CBFα AML. In CBFα AML, kinase-associated mutation, when combined together, exerted a strong negative effect on survival (hazard ratio=1.617 (95% confidence interval: 1.048–2.495), *P*=0.030 and hazard ratio=1.759 (95% confidence interval: 1.005–3.079), *P*=0.048 for OS and DFS, respectively), which was further proved by multivariate analysis.

In conclusion, CBF AML is a heterogeneous disease, whose clinical behavior and treatment outcome is strongly dependent on additional gene mutations, especially for class I mutations.^13^ Further improvement of the prognosis of the disease should corporate TKI in the standard treatment algorithm, and several groups reported a promising result.^14^ This strategy may especially benefit Chinese CBF leukemia patients, new clinical trial integrating homoharritonin,^15^ which is proved to improve the survival of AML in Chinese population, and TKI are ongoing (ChiCTR-IPR-15006862).

## Figures and Tables

**Figure 1:**
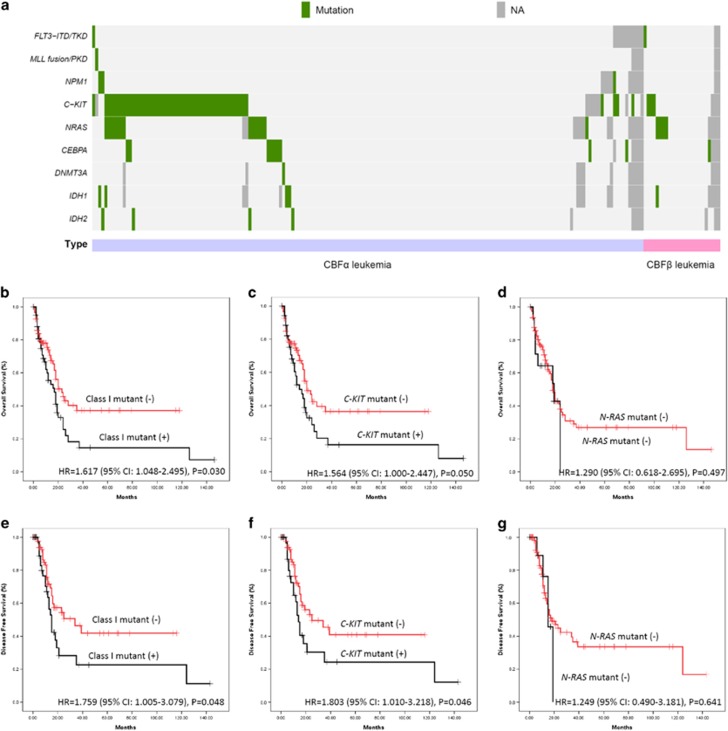
Additional mutations in CBF leukemia. (**a**) Distribution of additional mutations in CBF leukemia. (**b**–**d**) OS for the patients with different status of class I, *C-KIT* and *N-RAS* mutation. (**e**–**g**) DFS for the patients with different status of class I, *C-KIT* and *N-RAS* mutation.

**Table 1:** Clinical characteristics of 205 CBF AML patients

*Characteristics*	*CBFα leukemia*	*CBFβ leukemia*
*Gender no. (%)*		
Male	80 (44.4)	9 (36)
Female	100 (55.6)	16 (64)
Median age, years	34±19.1	40±22.2
Median WBC count,10^9^/l	8.95	48.1
Range	(0.8–177.9)	(3.8–140.0)
Median BM blasts, %	56	78
		
*FAB subtype no. (%)*		
M2v	143	
M4	27	
M4eo		25
M5	5	
Not classified	5	

Abbreviations: BM, bone marrow; M4eo, M4 with eosinophilia; M2v, M2 variant; WBC, white blood cell.
