# Supplementary material for: Kinase-associated gene mutation pattern and clinical relevance in 205 patients with core binding factor leukemias
Source: Blood Cancer J. 2016 Nov 11;6(11):e494–. doi: 10.1038/bcj.2016.107 (PMC5148054; doi:10.1038/bcj.2016.107)
Supplement: Supplementary Information [file bcj2016107x1.docx]

**Supplementary Material**

**Kinase associated gene mutation pattern and clinical relevance in 205 patients with core binding factor leukemias**

**Figure 1. Kaplan-Meier curves for OS and DFS for the patients with CBFα and CBFβ leukemias (A)** OS**, (B)** DFS

**Figure 1**

**B**

**A**


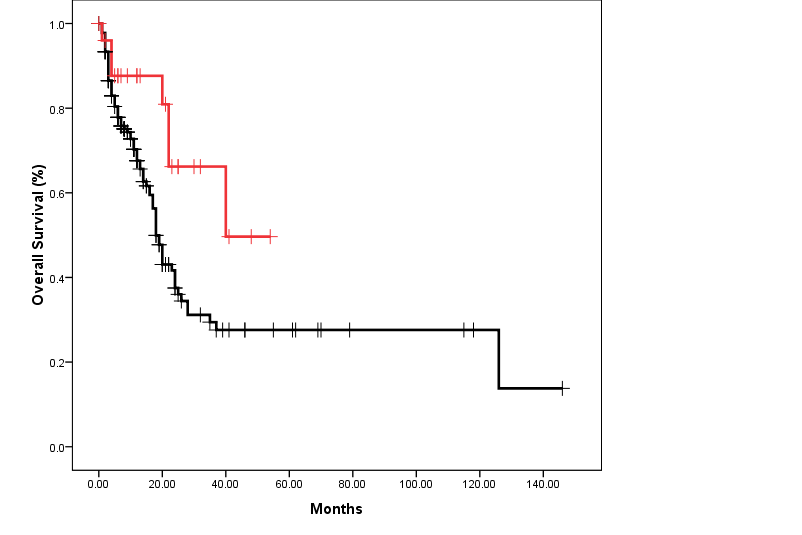

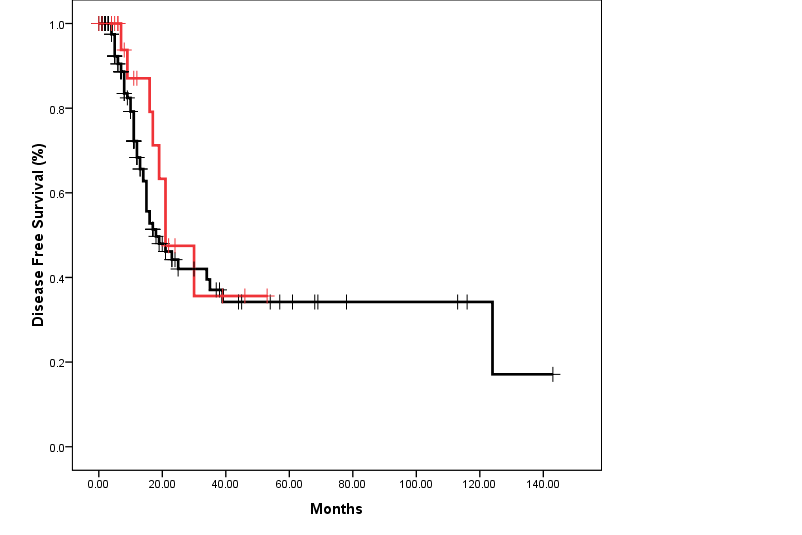


HR=0.744 (95% CI:0.352-1.571), P=0.438

HR=0.425 (95% CI:0.196-0.920), P=0.030

CBFα leukemia (N=138)

CBFβ leukemia (N=22)

CBFα leukemia (N=180)

CBFβ leukemia (N=25)

**Supplementary Tables**

**Table 1.** Gene mutations of the patients

| **Gene Mutations** | **CBFα leukemias** | **CBFβ leukemias** |
| --- | --- | --- |
| ***FLT3-ITD/TKD*** |  |  |
| Mutated | 1(0.6) | 1(4.3) |
| Not Mutated | 169(99.4) | 22(95.7) |
| NA | 10 | 2 |
| ***NRAS*** |  |  |
| Mutated | 14(8.4) | 4(19.0) |
| Not mutated | 154(91.6) | 17(81.0) |
| NA | 13 | 4 |
| ***C-KIT*** |  |  |
| Mutated | 52(30.2) | 3(13.6) |
| Not mutated | 120(69.8) | 19(86.4) |
| NA | 8 | 3 |
| ***MLL fusion /PKD*** |  |  |
| Mutated | 1(0.6) | 0 |
| Not mutated | 175(99.4) | 23 |
| NA | 4 | 2 |
| ***NPM1*** |  |  |
| Mutated | 3(1.6) | 0 |
| Not mutated | 168(98.4) | 23 |
| NA | 9 | 2 |
| ***CEBPA*** |  |  |
| Mutated | 9(5.1) | 1(4.5) |
| Not mutated | 166(94.9) | 21(95.5) |
| NA | 5 | 3 |
| ***DNMT3A*** |  |  |
| Mutated | 1(0.6) | 0 |
| Not mutated | 168(99.4) | 22 |
| NA | 11 | 3 |
| ***IDH1*** |  |  |
| Mutated | 4(2.4) | 1(4.8) |
| Not mutated | 162(97.6) | 20(95.2) |
| NA | 4 | 4 |
| ***IDH2*** |  |  |
| Mutated | 4(2.3) | 0 |
| Not mutated | 171(97.7) | 22 |
| NA | 5 | 3 |

NA, not available or failed

**Table 2.** Reported incidence of *C-KIT*, *FLT3*, *N-RAS* mutations in CBF AML patients

|  | ***C-KIT***  n(%) | ***FLT3***  n(%) | ***NRAS***  n(%) |
| --- | --- | --- | --- |
| **CBFα leukemias** |  |  |  |
| Our work | 52/172(30) | 1/170(<1) | 14/167(8) |
| N Boissel et al.([1](#_ENREF_1)) | 6/50(12) | 5/56(9) | 2/50(4) |
| M Tokumasu et al.([2](#_ENREF_2)) | 46/107(43) | / | / |
| C Allen et al.([3](#_ENREF_3)) | 46/199(23) | 27/199(14) | 25/199(13) |
| Eric Jourdan et al.([4](#_ENREF_4)) | 22/96(23) | 10/96(10) | 13/96(14) |
| Nicolas et al.([5](#_ENREF_5)) | 42/106(40) | 15/106(14) | 24/106(23) |
| Sang Hyuk Park et al.([6](#_ENREF_6)) | 7/71(10) | 1/71(1) |  |
| **CBFβ leukemias** |  |  |  |
| Our work | 3/22(13) | 1/23(4) | 4/21(19) |
| N Boissel et al.([1](#_ENREF_1)) | 10/46(22) | 3/47(6) | 15/47(32) |
| C Allen et al.([3](#_ENREF_3)) | 54/155(35) | 28/155(18) | 41/155(26) |
| Eric Jourdan et al.([4](#_ENREF_4)) | 18/102(18) | 20/102(20) | 28/102(27) |
| M Tokumasu et al.([2](#_ENREF_2)) | 12/31(39) | / | / |
| Elli et al.([7](#_ENREF_7)) | 12/81(15) | 12/81(15) | 43/81(53) |
| Nicolas et al.([5](#_ENREF_5)) | 36/109(33) | 26/109(24) | 42/109(39) |
| Peter et al.([8](#_ENREF_8)) | 65/176(37) | 30/176(17) | 78/176(44) |
| Sang Hyuk Park et al.([6](#_ENREF_6)) | 3/21(14) | 2/21(10) | / |

/:No data

**Table 3.** Gene mutations and clinical aspects

| **Gene mutations** | **Gender** | | **Median age，y** | **Median WBC count,10^9^/L**  **(range)** | **Median BM blasts，%**  **（range）** |
| --- | --- | --- | --- | --- | --- |
|  | **No.(%)** | |  |  |  |
|  | **Male** | **Female** |  |  |  |
| ***C-KIT*** |  | | | | |
| Mutated | 30 | 25 | 34.0±19.0 | 10.9(0.8-137.9) | 67(21.5-90.0) |
| Not mutated | 77 | 62 | 34.0±19.5 | 9.8(1-177.9) | 54(23.5-91.0) |
| P value | 0.915 | | 0.796 | 0.602 | <0.001 |
| ***N-RAS*** |  | | | | |
| Mutated | 9 | 9 | 31.5±22.5 | 20.9(3.5-67.1) | 67(28.0-85.0) |
| Not mutated | 96 | 74 | 34.0±19.0 | 9.1(0.8-177.9) | 51(23.5-.89.0) |
| P value | 0.599 | | 0.980 | 0.168 | 0.099 |
| **Class I** |  | | | | |
| Mutated | 30 | 25 | 34.0±19.0 | 10.9(0.8-137.9) | 67(21.5-90.0) |
| Not mutated | 66 | 47 | 33.0±18.8 | 7.9(1.0-177.9) | 51(23.5-.89.0) |
| P value | 0.635 | | 0.576 | 0.908 | <0.001 |

No, number of patients

Class I refers to patients that have *FLT3*-ITD/TKD, *C-KIT* or *NRAS* mutations

**Table 4.** The relationship between gene mutations status and CR rate

| **Gene mutations** | **CBFα leukemias** | **CBFβ leukemias** |
| --- | --- | --- |
|  | **CR No.(%)** | **CR No.(%)** |
| ***C-KIT*** |  |  |
| Mutated | 37/52 (71.2) | 2/3 (66.7) |
| Not mutated | 94/120 (78.3) | 17/19 (89.5) |
| P value | 0.334 | .296 |
| ***N-RAS*** |  |  |
| Mutated | 9/14 (64.3) | 4/4 (100) |
| Not mutated | 121/154 (79.0) | 14/17 (82.4) |
| P value | 0.313 | .376 |
| ***FLT3*-ITD/TKD** |  |  |
| Mutated | 0/1 | 1/1 (100) |
| Not mutated | 130/169 (77.4) | 19/22 (86.4) |
| P value | 0.235 | .157 |
| **Class I** |  |  |
| Mutated | 42/59 (72.5) | 7/8 (87.5) |
| Not mutated | 95/120 (79.2) | 12/14 (85.7) |
| P value | 0.263 | 1.000 |

No, number of patients

CR, complete remission

**Table 5.** Multivariate analysis of clinical and molecular variables for OS and DFS in CBFα leukemia

| **Variables** | **OS** | | **DFS** | |
| --- | --- | --- | --- | --- |
|  | **P** | **OR(95% CI)** | **P** | **OR(95% CI)** |
| Age | 0.086 | 1.010  (0.999-1.022) | NS | - |
| WBC count | NS | - | NS | - |
| Blast | NS | - | NS | - |
| Class I mutations | 0.041 | 1.575  (1.020-2.434) | NS | - |

**REFERNCES**

1. Boissel N, Leroy H, Brethon B, Philippe N, de Botton S, Auvrignon A, et al. Incidence and prognostic impact of c-Kit, FLT3, and Ras gene mutations in core binding factor acute myeloid leukemia (CBF-AML). Leukemia. 2006 Jun;20(6):965-70.

2. Tokumasu M, Murata C, Shimada A, Ohki K, Hayashi Y, Saito AM, et al. Adverse prognostic impact of KIT mutations in childhood CBF-AML: the results of the Japanese Pediatric Leukemia/Lymphoma Study Group AML-05 trial. Leukemia. 2015 Dec;29(12):2438-41.

3. Allen C, Hills RK, Lamb K, Evans C, Tinsley S, Sellar R, et al. The importance of relative mutant level for evaluating impact on outcome of KIT, FLT3 and CBL mutations in core-binding factor acute myeloid leukemia. Leukemia. 2013 Sep;27(9):1891-901.

4. Jourdan E, Boissel N, Chevret S, Delabesse E, Renneville A, Cornillet P, et al. Prospective evaluation of gene mutations and minimal residual disease in patients with core binding factor acute myeloid leukemia. Blood. 2013 Mar 21;121(12):2213-23.

5. Duployez N, Marceau-Renaut A, Boissel N, Petit A, Bucci M, Geffroy S, et al. Comprehensive mutational profiling of core binding factor acute myeloid leukemia. Blood. 2016 May 19;127(20):2451-9.

6. Park SH, Lee HJ, Kim I-S, Kang J-E, Lee EY, Kim H-J, et al. Incidences and Prognostic Impact ofc-KIT,WT1,CEBPA, andCBLMutations, and Mutations Associated With Epigenetic Modification in Core Binding Factor Acute Myeloid Leukemia: A Multicenter Study in a Korean Population. Annals of Laboratory Medicine. 2015;35(3):288.

7. Papaemmanuil E, Gerstung M, Bullinger L, Gaidzik VI, Paschka P, Roberts ND, et al. Genomic Classification and Prognosis in Acute Myeloid Leukemia. The New England journal of medicine. 2016 Jun 9;374(23):2209-21.

8. Paschka P, Du J, Schlenk RF, Gaidzik VI, Bullinger L, Corbacioglu A, et al. Secondary genetic lesions in acute myeloid leukemia with inv(16) or t(16;16): a study of the German-Austrian AML Study Group (AMLSG). Blood. 2013 Jan 3;121(1):170-7.
